# Quantitative Analysis of Perfluorooctanoic Acid and Perfluorooctane Sulfonate Mixtures Using 3D Surface-Enhanced Raman Spectroscopy with Single-Photon Detection

**DOI:** 10.3390/s26216865

**Published:** 2026-10-30

**Authors:** Tianhang Huo, Yehong Li, Santosh Kumar, Silvana Andreescu, Yu-Ping Huang, Henry Du

**Affiliations:** 1 Department of Chemical Engineering and Materials Science, Stevens Institute of Technology, 1 Castle Point Terrace, Hoboken, NJ 07030, USA; 2 Department of Physics, Stevens Institute of Technology, 1 Castle Point Terrace, Hoboken, NJ 07030, USA; 3 Department of Chemistry and Biochemistry, Clarkson University, 8 Clarkson Ave, Potsdam, NY 13699, USA

**Keywords:** per- and polyfluoroalkyl substances, surface-enhanced Raman spectroscopy, single-photon detection, plasmonic nanostructures, multivariate regression, environmental nanosensing

## Abstract

Per- and polyfluoroalkyl substances (PFASs) are persistent environmental contaminants that commonly occur as mixtures, yet their weak Raman scattering and overlapping vibrational features make direct optical quantification challenging. Here, we develop a plasmonic nanosensing platform that integrates three-dimensional Ag nanoparticle surface-enhanced Raman spectroscopy (SERS) substrates with single-photon Raman readout for quantitative analysis of binary perfluorooctanoic acid (PFOA)/perfluorooctane sulfonate (PFOS) mixtures. The multilayer silver nanoparticle (AgNP) architecture provides dense electromagnetic hot spots to amplify weak PFAS signatures, while photon-counting detection improves the measurement of low-intensity Raman features. Mixed spectra obtained from a 4 × 4 concentration matrix spanning $10^{-6}$ to $10^{-15}$ M exhibited substantial peak overlap, band broadening, and concentration-dependent spectral contour changes, highlighting the limitations of single-band calibration. Full-spectrum multivariate regression was therefore used to extract composition-dependent information from the coupled SERS response. Under nested cross-validation with replicates grouped by mixture composition, both partial least squares regression and linear support vector regression captured the main concentration-dependent spectral variation, achieving $R^{2}$ values of approximately 0.81–0.87 across the two analytes and identifying experimental concentration levels within one adjacent level after nearest-level assignment. These results demonstrate that coupling engineered plasmonic AgNP substrates with single-photon Raman detection enables mixture-resolved PFAS quantification from weak and overlapping SERS signatures, providing a nanomaterial-enabled framework for environmental molecular sensing.

## 1. Introduction

Per- and polyfluoroalkyl substances (PFASs) are a diverse group of synthetic organofluorine compounds containing fully fluorinated carbon moieties. Their widespread environmental occurrence, resistance to degradation, and potential risks to human and ecological health have attracted sustained attention. Environmental releases are associated with fluorochemical manufacturing, historical use of aqueous film-forming firefighting foams, treated textiles and paper products, and wastewater and waste-disposal pathways. Among PFASs, perfluorooctanoic acid (PFOA) and perfluorooctane sulfonate (PFOS) are extensively investigated because of their historical production, frequent detection, and toxicological relevance. Under typical environmental aqueous conditions, they occur predominantly as the anions ${\mathrm{CF}}_{3}{\left({\mathrm{CF}}_{2}\right)}_{6}{\mathrm{COO}}^{-}$ and ${\mathrm{CF}}_{3}{\left({\mathrm{CF}}_{2}\right)}_{7}{\mathrm{SO}}_{3}^{-}$, respectively. Their different terminal groups, carboxylate for PFOA and sulfonate for PFOS, influence their interfacial interactions and vibrational signatures. Exposure to certain PFASs has been associated with adverse immune, developmental, hepatic, and other health effects [1,2,3,4,5,6,7,8,9]. However, no single aqueous concentration universally defines toxicity, because health risks depend on the compound, exposure route and duration, and health endpoint. As a regulatory benchmark, the U.S. Environmental Protection Agency’s 2024 National Primary Drinking Water Regulation established individual maximum contaminant levels of $4\;\mathrm{ng}\;L^{-1}$ for PFOA and PFOS [10]. These regulatory limits, rather than universal thresholds for the onset of toxicity, underscore the need for sensitive and selective PFAS determination.

At present, PFAS analysis is dominated by chromatographic and mass spectrometric approaches, particularly liquid chromatography–tandem mass spectrometry (LC-MS/MS), which remains the benchmark for sensitive and selective determination across a range of matrices [11,12,13,14,15]. Complementary approaches, including total oxidizable precursor assays, organofluorine-based screening methods, and electrochemical sensing, have also been explored for broader PFAS characterization and detection [16,17,18,19]. Fourier-transform infrared spectroscopy (FTIR) provides complementary structural information through vibrations associated with the carboxylate and sulfonate groups of PFOA and PFOS, respectively. For example, Gao and Chorover used attenuated total reflection FTIR spectroscopy to identify distinct binding mechanisms of these compounds at hematite surfaces [20]. However, structural differentiation does not by itself establish reliable quantification of trace-level aqueous mixtures. Existing analytical approaches also face method-specific limitations, including sophisticated instrumentation, laborious workflows, indirect measurement of aggregate PFAS content, or limited field adaptability [11,13,16,17,18,19]. These limitations motivate the development of rapid, label-free, and potentially more deployable approaches, including surface-enhanced Raman spectroscopy (SERS) combined with single-photon detection and multivariate analysis to acquire weak vibrational signals and resolve overlapping analyte contributions.

Raman spectroscopy is attractive in this context because it offers molecularly specific, label-free analysis with little or no sample preparation [21,22]. Nevertheless, direct Raman measurement of PFAS is intrinsically difficult because these compounds lack strong chromophores, exhibit low polarizability, and generate weak Raman scattering [16,17,21,22]. Surface-enhanced Raman spectroscopy (SERS) provides a practical route to overcome this limitation by exploiting plasmonic nanostructures to amplify Raman scattering by many orders of magnitude. Owing to its high sensitivity and molecular specificity, SERS has become an important analytical tool in environmental monitoring, food safety, biomedical detection, and chemical sensing [23,24,25,26,27,28,29,30].

Despite this general progress, PFAS-oriented SERS studies remain comparatively limited. Existing reports have largely focused on indirect detection schemes, functionalized substrates, dry-state measurements, or single-compound demonstrations [31,32,33,34]. For example, PFAS detection has been reported using indicator-assisted Raman strategies, modified plasmonic interfaces, and selective nanostructured substrates, while only a small number of studies have begun to address quantitative or mixture-related Raman analysis [35,36,37]. These advances are important, but they do not yet fully address a key analytical reality: environmentally relevant PFAS contamination rarely occurs as a single analyte in isolation. Instead, mixtures of coexisting PFAS species are more typical, and such mixtures generate more complex spectral and interfacial behavior than single-component systems [1,2,3,4,5,11,12,13,14,15,31,32,33,34,35,36,37].

Quantitative analysis of PFAS mixtures is therefore more challenging than simply detecting whether PFASs are present. In binary or multicomponent systems, partially overlapping spectral features, different Raman response factors, competitive adsorption on SERS-active surfaces, and hotspot heterogeneity can all complicate direct concentration readout [26,35,36,37]. Under such conditions, single-peak analysis is often insufficient. Multivariate methods are consequently more suitable because they can extract composition-dependent information from the full spectral profile rather than from isolated bands alone. In Raman analysis, chemometric methods such as partial least squares (PLS) regression are well established for quantitative treatment of complex, multicomponent datasets and are particularly valuable when weak but systematic spectral variation must be translated into concentration information [21,22].

In addition to substrate design, detector performance becomes a central analytical factor when weak Raman signals must support not only detection but also quantitative resolution of PFAS mixtures. This point is especially important for PFASs because the spectral information of interest is often weak, and the success of mixture quantification depends on whether subtle but chemically meaningful variation can be recovered with adequate signal-to-noise ratio. Single-photon detection is therefore particularly attractive in this setting. Photon-counting and SPAD-based detection strategies have been shown to improve sensitivity for low-intensity optical signals and, in Raman applications, can support efficient acquisition of weak spectral responses [38,39,40,41,42]. Time-gated or time-resolved single-photon detection can additionally suppress fluorescence background, further improving the analytical utility of spectra acquired under challenging conditions [39,40,41,42]. Rather than serving only as an alternative detector format, single-photon Raman detection can thus function as an enabling readout strategy for extracting concentration-relevant information from weak SERS measurements.

Building on these considerations, the present study focuses on the quantitative analysis of PFOA/PFOS mixtures using a 3D SERS substrate coupled with single-photon Raman detection. Here, the role of the 3D SERS architecture is to enhance intrinsically weak PFAS Raman responses, while the role of the single-photon detector is to increase the analytical value of those enhanced spectra for concentration prediction. By integrating this measurement platform with multivariate analysis, we examine whether composition-dependent Raman variation from binary PFAS mixtures can be resolved quantitatively. In this way, the work shifts the emphasis of PFAS Raman sensing from single-analyte demonstration toward mixture-resolved quantification with greater relevance to practical environmental analysis [38,43].

## 2. Materials and Methods

### 2.1. Materials

The reagents used included sodium citrate dihydrate (Na_3_C_6_H_5_O_7_·2H_2_O, 99%), silver nitrate (AgNO_3_, 99%), poly(allylamine hydrochloride) (PAH, average molecular weight 15,000), sodium hydroxide (NaOH, 97%), and rhodamine 6G (C_28_H_31_N_2_O_3_Cl, 99%), all purchased from Sigma-Aldrich (St. Louis, MO, USA). Silicon wafers were purchased from MSE Supplies (Tucson, AZ, USA). Perfluorooctanoic acid (PFOA, CF_3_(CF_2_)_6_COOH, 95%; CAS No. 335-67-1; Cat. No. 171468) was purchased from Sigma-Aldrich (St. Louis, MO, USA). Perfluorooctanesulfonic acid (PFOS, CF_3_(CF_2_)_7_SO_3_H, 98.7+%; CAS No. 1763-23-1; Cat. No. MSK191968) was purchased from A Chemtek (Woburn, MA, USA). Both PFAS reagents were supplied as solids in the free-acid form.

### 2.2. SERS Substrate Preparation

AgNP colloids and multilayered AgNP SERS substrates were prepared following our previously reported procedure [36]. Briefly, AgNP colloids were synthesized by citrate reduction of AgNO_3_, yielding nanoparticles with an average diameter of 40 ± 5 nm, a ζ-potential of −40±5 mV, and a localized surface plasmon resonance peak at 406 nm. For substrate fabrication, 10 mm × 10 mm silicon wafers with a native oxide layer were immersed in poly(allylamine hydrochloride) (PAH) solution (0.2 mg/mL, pH 9) for 20 min and rinsed with Milli-Q water. The positively charged PAH layer promoted electrostatic assembly of the negatively charged AgNPs. The substrates were subsequently immersed in the AgNP colloidal solution for 8 h in the dark, followed by rinsing with Milli-Q water at pH 4.5. A total of eight PAH/AgNP deposition cycles were performed to obtain the eight-layer AgNP substrates used in this study. The layer number denotes the number of assembly cycles.

### 2.3. Single-Photon Raman Measurement

**Figure 1:**
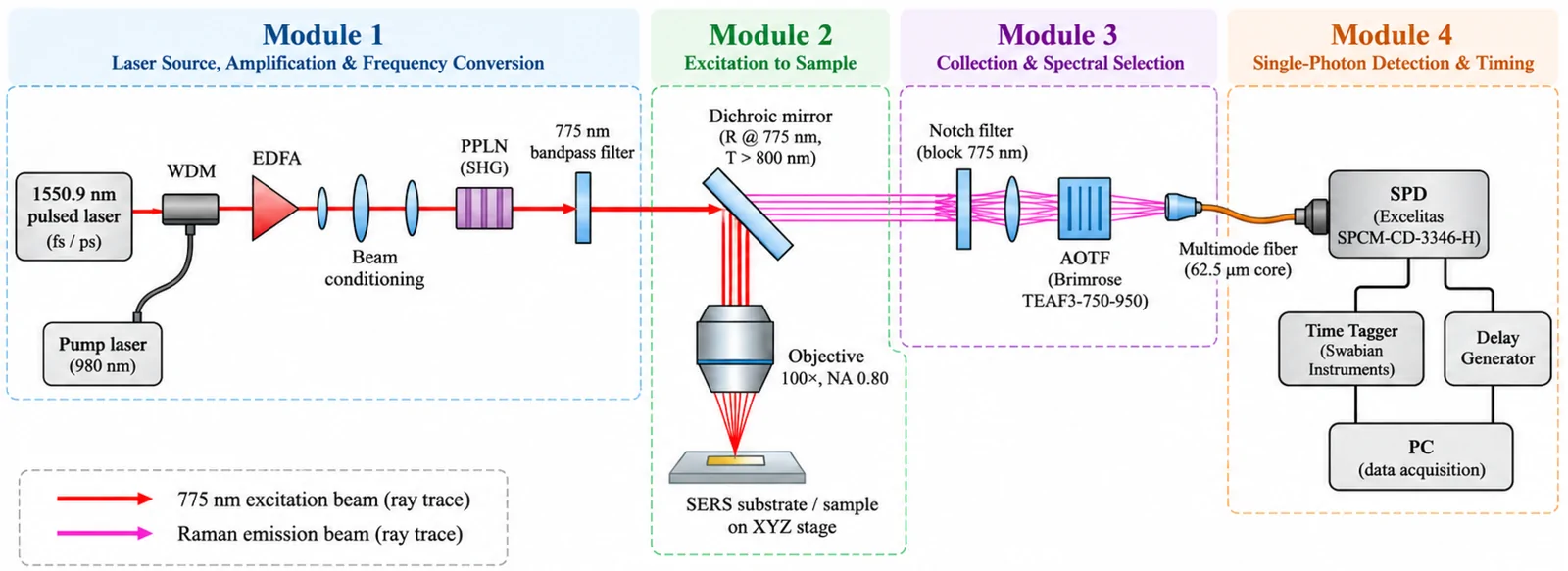
Schematic illustration of the SERS–single-photon Raman detection platform. A mode-locked laser operating at 1550.9 nm generates second-harmonic excitation at approximately 775 nm. Raman photons from the SERS substrate are spectrally selected by an acousto-optic tunable filter (AOTF) and detected using a single-photon detector (SPD).

The SERS–SPD platform has been described previously [36,43]; only a brief summary is provided here. The acquisition parameters used for the present mixture measurements are summarized in Supplementary Section S2. In this system, Raman photons are spectrally selected by an acousto-optic tunable filter (AOTF) and subsequently recorded by a single-photon detector. The output from a 1550.9 nm pulsed laser source (CALMAR Laser (Palo Alto, CA, USA)) was wavelength-selected using a wavelength-division multiplexer (WDM), amplified using an erbium-doped fiber amplifier (EDFA), and converted to a 775 nm pulsed excitation beam through second-harmonic generation in a nonlinear crystal. The excitation beam was directed through free space into an objective lens (100×, NA 0.8; Nikon CFI 60 LU Plan Epi ELWD (Nikon, Tokyo, Japan)) and focused onto the sample surface. Backscattered light was collected through the same objective and routed through a dichroic mirror, a notch filter, and the AOTF (Brimrose Corp. (Sparks, MD, USA); tellurium dioxide noncollinear crystal, TEAF3-750-950). The selected Raman signal was then coupled into a multimode fiber and detected by a single-photon detector (Excelitas Technologies (Waltham, MA, USA) SPCM-CD-3346-H), with a dark count rate of approximately 50 Hz, a dead time of approximately 25 ns, and a timing resolution of approximately 350 ps. The time tagger (Time Tagger Ultra, Swabian Instruments, Stuttgart, Germany) recorded photon arrival times relative to the laser synchronization signal. The delay generator adjusted the synchronization delay to align the detected signal with the acquisition window. Software time gating was applied by integrating photon counts within a selected temporal window around the synchronized signal peak, thereby excluding background counts outside this window. Data processing used MATLAB R2020a Update 1 (version 9.8.0.1359463; MathWorks, Natick, MA, USA). A schematic illustration of the system is presented in Figure 1. Solvent blanks were deposited onto the SERS substrates using the same volume as the PFAS samples and measured in the liquid state under identical acquisition conditions.

### 2.4. Spectral Preprocessing and Quantitative Analysis

Owing to the practical difficulty of experimentally measuring mixed samples over a continuous concentration range, four representative concentration levels were selected for both PFOA and PFOS to provide broad coverage of the target detection window. On this basis, a 4 × 4 concentration matrix was constructed using concentrations of $10^{-6}$, $10^{-9}$, $10^{-12}$ and $10^{-15}$ M for each analyte, yielding 16 mixed samples in total. Following our previously reported procedure [36], 1 μL of each PFOA/PFOS mixture was drop-cast onto each of three independently prepared SERS substrates. Measurements were initiated after deposition without a separate adsorption–incubation step, and spectra were acquired while the droplet remained liquid, without drying. Five spectra were collected at different positions on each substrate, yielding 15 spectra per concentration combination and 240 spectra across the 16 combinations.

Prior to model construction, all spectra were preprocessed by background correction to minimize variations arising from residual baseline contributions. The preprocessed spectra were then matched with their corresponding concentration labels. To account for the wide dynamic range investigated, the concentrations of PFOA and PFOS were expressed on a logarithmic scale for model training.

For assessment of spectral quality, the signal-to-noise ratio (S/N) was estimated from the averaged spectra as

(1) $$S/N=\frac{I_{\mathrm{peak}}-I_{\mathrm{baseline}}}{\sigma_{\mathrm{noise}}},$$

where $I_{\mathrm{peak}}$ is the intensity at the selected spectral feature, $I_{\mathrm{baseline}}$ is the local baseline at the same Raman shift, and $\sigma_{\mathrm{noise}}$ is the standard deviation of the linearly detrended signal within 1450–1650 cm^−1^. The calculation windows and estimated S/N values are provided in Table S1.

Supervised regression models were established to simultaneously predict the concentrations of PFOA and PFOS from the measured spectra. Partial least squares (PLS) regression was selected as the primary quantitative model because it is well suited to highly collinear spectral data and enables extraction of latent variables that maximize the covariance between spectral features and analyte concentrations. The input matrix, X, consisted of the preprocessed Raman spectra, while the response matrix, *Y*, contained the log-transformed concentrations of PFOA and PFOS for each sample.

Model performance was evaluated using nested GroupKFold cross-validation, with five outer folds for performance evaluation and four inner folds for hyperparameter selection. Groups were defined by the PFOA/PFOS concentration combinations, with all replicate spectra from each combination kept together in both validation loops. Thus, each outer test set contained concentration combinations absent from its corresponding training set, evaluating prediction for held-out combinations within the investigated concentration grid. Details of model training, hyperparameter selection, and the parameters selected in each outer fold are provided in Supplementary Section S1 and Table S2.

The models predicted continuous values of $\log_{10}\left(C/M\right)$. The coefficient of determination ($R^{2}$), RMSE, and MAE were calculated from the pooled outer-fold held-out predictions and their reference values, without rounding or assignment to discrete concentration levels.

Separately, concentration-level accuracy was evaluated by assigning each prediction to the nearest of the four tested log-concentration levels (−15, −12, −9, and −6), indexed as 0–3. Exact-level accuracy required the predicted and reference indices to match, whereas ±1-level accuracy allowed an index difference of at most one. This latter criterion concerns discrete concentration levels, not a continuous error within one order of magnitude.

## 3. Results and Discussion

### 3.1. Spectral Characteristics of PFOA/PFOS Mixtures

**Figure 2:**
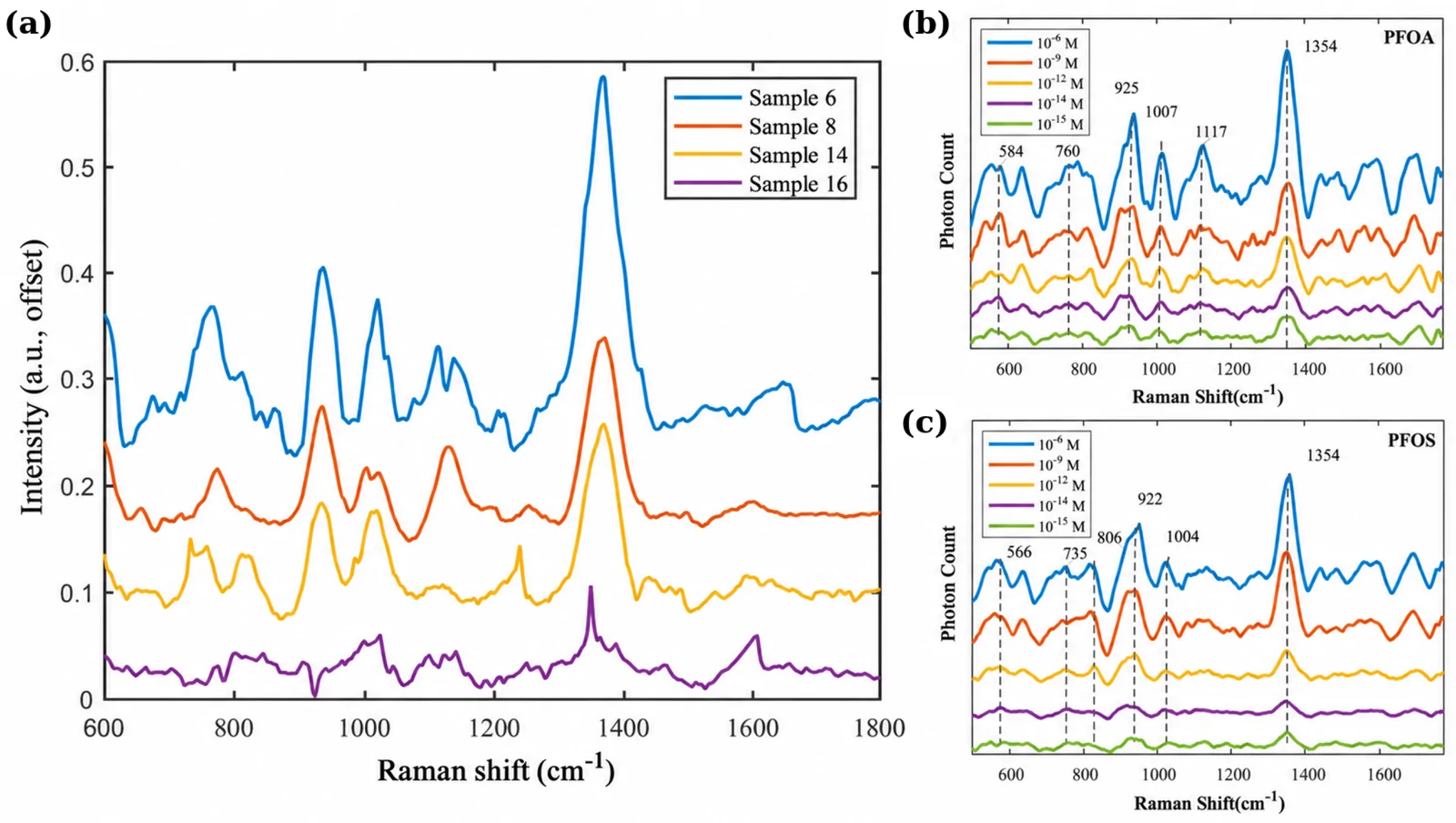
Raman spectra of PFOA/PFOS mixtures and individual components. (**a**) Raman spectra of selected PFOA/PFOS mixtures; (**b**) PFOA and (**c**) PFOS spectra measured at different concentrations.

To analyze the spectral behavior of the binary system, it is first necessary to consider the Raman features of the individual components. As shown in Figure 2, PFOA exhibits characteristic bands at approximately 584, 760, 925, 1007, 1117, and 1354 cm^−1^, while PFOS shows characteristic features near 566, 735, 806, 922, 1004, and 1354 cm^−1^. Based on previously reported assignments, the PFOA band near 584 cm^−1^ includes contributions from CF_2_ deformation, while the bands near 760 and 1117 cm^−1^ involve coupled carboxylic-headgroup/CF_2_ motions and C–C skeletal/CF_2_ motions, respectively. For PFOS, the bands near 566 and 735 cm^−1^ have been associated with coupled sulfonic-headgroup/CF_2_ deformation and CF_2_ deformation, respectively. The features near 806 and 922 cm^−1^ have been associated with headgroup-related coupled motions, whereas the band near 1004 cm^−1^ involves C–C skeletal and CF_2_ motions [36]. CF_3_ vibrations reported in the 1350–1370 cm^−1^ region may contribute to the response near 1354 cm^−1^ in both analytes [44]. These assignments are tentative because molecular ionization, conformation, and surface interactions can alter the observed vibrational features; the PFOA bands near 925 and 1007 cm^−1^ are retained as observed features without definitive mode assignments. The closely spaced bands near 925/922 and 1007/1004 cm^−1^, together with the shared response near 1354 cm^−1^, limit discrimination using individual peak positions. Consequently, analysis of the combined spectral fingerprint is needed to extract composition-dependent information when both compounds are present. To assess the low-concentration spectral response, Figure S1 compares the solvent blank with individual PFOA and PFOS spectra at $10^{-15}$ M. Estimated S/N values were 10.25–16.64 for PFOA and 6.35–10.76 for PFOS (Table S1). These results characterize the spectral response at the lowest nominal concentration investigated.

The Raman spectra of the PFOA/PFOS mixtures are shown in Figure 2. Compared with the spectra of the pure components, the mixture spectra exhibit a more complex profile, reflecting the combined contribution of both analytes. Characteristic information from PFOA and PFOS is still retained in the mixed spectra; however, the corresponding spectral features are no longer fully independent. In several regions, bands attributed to the two species appear at similar Raman shifts and therefore contribute to overlapping composite features. This is particularly evident for peaks in the low-wavenumber region and for the strong band near 1354 cm^−1^, where both analytes show prominent responses in the single-component spectra. Consequently, the spectrum of a mixture cannot be interpreted simply as a set of isolated peaks uniquely representing each compound.

In the mixed spectra, the main spectral features can still be related qualitatively to the characteristic bands observed for the individual compounds. However, the correspondence is not one-to-one, because several bands from PFOA and PFOS are positioned closely and contribute simultaneously to the observed mixture response. Accordingly, spectral variation among different concentration combinations is reflected not only in peak intensity changes, but also in changes in peak contour, relative prominence, and local line shape. This further supports the view that the analytical information is distributed across the spectrum rather than confined to a small number of isolated bands.

In addition to peak overlap, the mixture spectra also show band broadening and reduced separation between neighboring features. For peaks located at close Raman shifts, the corresponding signals may merge into a broader envelope rather than appear as clearly resolved maxima. Under such conditions, the apparent peak shape is strongly influenced by the relative contributions of both analytes. This effect becomes more pronounced when the concentrations of PFOA and PFOS differ substantially. When one component is present at a higher concentration, its signal may dominate the local spectral profile, while the characteristic feature of the lower-concentration component may only appear as a weak shoulder or may no longer be visually distinguishable. Therefore, the spectral expression of a given Raman band in the mixture is governed not only by its presence in the pure-component spectrum, but also by overlap with neighboring bands, relative band width, and the concentration ratio between the two analytes.

These observations have direct implications for quantitative analysis. In a binary PFAS system, the intensity of an individual peak cannot be assumed to arise from only one analyte, nor can it be expected to vary independently of the other component. As a result, univariate quantification based on a single Raman band is unlikely to provide a sufficiently robust description of the mixture composition. Instead, the mixture spectra should be treated as integrated multivariate signatures containing coupled information from both PFOA and PFOS. On this basis, multivariate analysis of the full spectral profile was employed in the following section to model the concentrations of PFOA and PFOS simultaneously.

### 3.2. Multivariate Analysis for Simultaneous Quantification of PFOA and PFOS

Single-band calibration can be effective when a spectrum contains isolated and concentration-sensitive bands. In the present PFOA/PFOS mixtures, several features overlap or appear as shoulders, depending on the relative analyte concentrations. These features may complicate the attribution of individual peak intensities to a specific analyte. Previous studies on quantitative Raman and SERS analysis have emphasized that univariate calibration can be effective in simple systems but may become less reliable when spectral overlap, matrix-dependent signal variation, or multiple analyte contributions are present [45,46,47]. In PFAS analysis, Rothstein et al. reported non-monotonic relationships between band intensity and concentration in their SERS system and discussed the resulting limitations of traditional peak-intensity calibration [44].

These considerations motivated the use of full-spectrum multivariate regression to extract concentration-related information distributed across the mixture spectra. The present binary-mixture study also serves as an initial step toward extending the platform to additional environmentally relevant PFASs, for which closely spaced bands may further complicate single-band analysis. The analysis focuses on the feasibility of full-spectrum prediction; its relative performance against single-band and selected-feature models remains to be evaluated under identical validation conditions.

PLSR was selected as the primary chemometric model because it is widely established for quantitative spectroscopic analysis of high-dimensional and collinear data [48,49]. PLSR projects the spectral matrix into latent variables that maximize covariance between spectral variation and analyte concentration. This approach is useful for SERS mixture analysis, where quantitative information may be distributed across overlapping spectral regions rather than confined to a single marker band. PLSR has been applied in quantitative SERS studies involving thymine, dipicolinic acid, neurotransmitters, pesticides, and multiplexed Raman-labelled DNA systems [50,51,52,53,54,55,56]. Linear SVR was included as a comparative model to evaluate whether an alternative regularized linear regression framework could improve prediction without the additional complexity of nonlinear kernels [47,57].

The models were evaluated using the composition-grouped five-fold cross-validation procedure described in Section 2.4. Table 1 summarizes performance based on continuous log-concentration predictions. For PFOA, PLSR achieved an $R^{2}$ of 0.813, an RMSE of 1.451, and an MAE of 1.223, whereas Linear SVR achieved an $R^{2}$ of 0.866, an RMSE of 1.229, and an MAE of 0.987. For PFOS, the models performed comparably, with $R^{2}$ values of 0.809 for PLSR and 0.806 for Linear SVR. These results indicate that Linear SVR improved PFOA prediction, while neither model consistently outperformed the other for PFOS.

**Figure 3:**
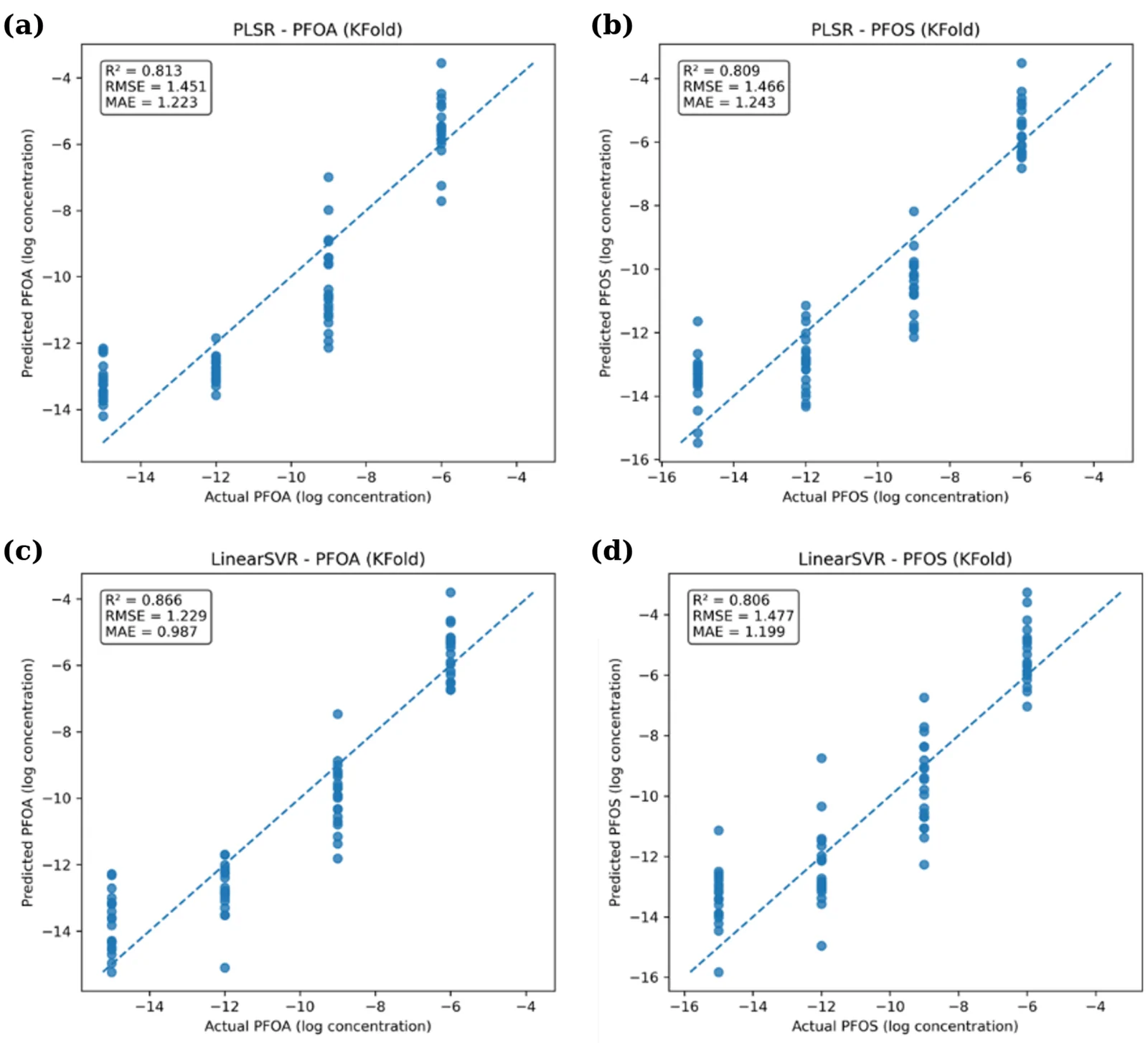
Predicted versus actual $\log_{10}$ concentrations of PFOA and PFOS: (**a**) PFOA predicted using PLSR; (**b**) PFOS predicted using PLSR; (**c**) PFOA predicted using Linear SVR; and (**d**) PFOS predicted using Linear SVR. Concentrations are expressed as $\log_{10}\left[C/\left(\mathrm{mol}\;L^{-1}\right)\right]$. Each point represents a held-out prediction. Dashed diagonal lines indicate perfect agreement between predicted and actual concentrations.

The predicted-versus-actual plots in Figure 3 illustrate model performance across the four concentration levels. For both analytes, predictions broadly followed the diagonal, indicating that PLSR and Linear SVR captured concentration-dependent information in the mixture spectra. Pronounced deviations occurred at the lowest concentration level, $10^{-15}$ M, where weak analyte-specific signals may be more susceptible to spectral noise, local variations in enhancement, and overlapping contributions from the coexisting analyte. Similar challenges have been discussed in multiplexed SERS analysis, motivating multivariate modelling rather than isolated peak analysis [45,46,55,57,58]. The observed trend supports concentration-dependent prediction, although the scatter indicates limited precision at individual concentration levels.

**Figure 4:**
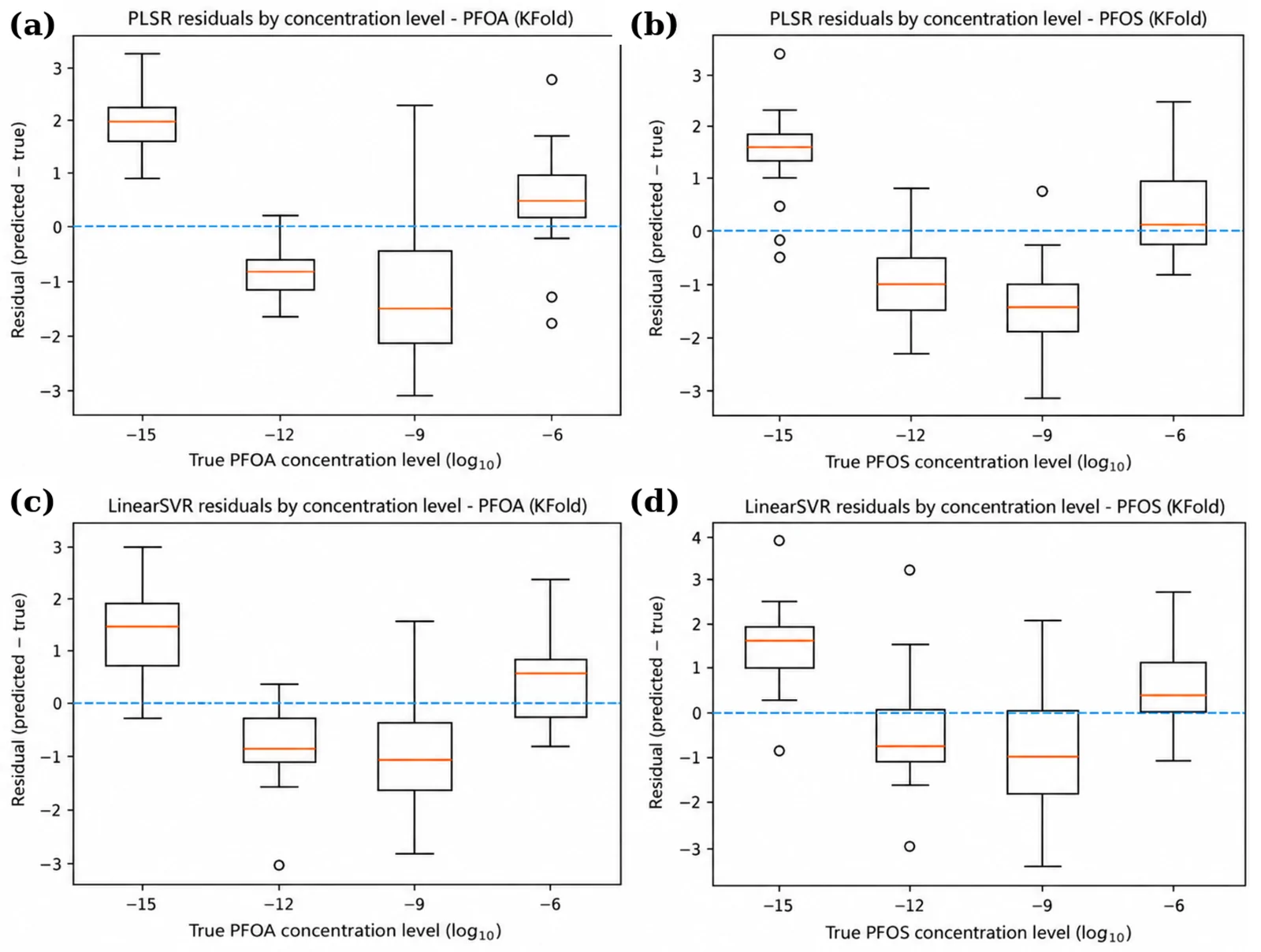
Distributions of prediction residuals at each actual concentration level: (**a**) PFOA predicted using PLSR; (**b**) PFOS predicted using PLSR; (**c**) PFOA predicted using Linear SVR; and (**d**) PFOS predicted using Linear SVR. Residuals are calculated as predicted minus actual $\log_{10}$ concentration. Positive and negative residuals indicate overestimation and underestimation, respectively. Boxes show the interquartile range, and horizontal lines within the boxes indicate medians. Dashed horizontal lines mark zero residual. Each concentration-level distribution includes predictions for mixtures containing different concentrations of the coexisting analyte.

The residual distributions in Figure 4 reveal concentration-dependent prediction errors. At the lowest concentration level, both models generally produced positive residuals, indicating overestimation. Residuals at intermediate levels, particularly $10^{-12}$ M and $10^{-9}$ M, were more frequently negative, indicating a tendency toward underestimation. At $10^{-9}$ M, some predictions deviated substantially from the reference concentration, contributing to the broad distributions in Figure 4a,d, consistent with the scatter in Figure 3a,d. At $10^{-6}$ M, residuals were closer to zero but retained a moderate spread. Linear SVR showed a narrower residual distribution for PFOA than PLSR, consistent with its lower RMSE and MAE, whereas the PFOS residual patterns were broadly comparable between the models.

At the lowest concentration, weak analyte-specific information and model regularization may shift predictions toward the central calibration range, whereas spectral overlap and limitations of the linear models may contribute to errors at intermediate levels. These possible contributions cannot be separated using the present dataset. The resulting concentration-dependent bias limits precise quantification, particularly at $10^{-15}$ M, despite the overall predictive performance.

These deviations may reflect measurement variability, heterogeneity in substrate preparation and local enhancement, or overlapping analyte contributions. Variations in substrate wetting and analyte adsorption during liquid-phase measurements may also affect molecular access to AgNP hot spots. The importance of spatial coupling between analyte carriers and plasmonic hot spots has been highlighted in magnetically controlled SERS platforms [59]. Furthermore, differences in adsorption kinetics can cause surface composition to differ from solution composition, as demonstrated in other multicomponent SERS systems [60]. These mechanisms are plausible contributors, but their individual effects were not established in the present experiments. Model limitations may also contribute to the systematic residual patterns. With only 16 independent concentration combinations, the dataset provides limited coverage of mixture variability, and large deviations substantially influence the reported residual distributions.

Taken together, the regression metrics and residual analysis support the feasibility of full-spectrum multivariate prediction within the investigated PFOA/PFOS concentration grid. PLSR provided an established chemometric baseline, while Linear SVR improved PFOA prediction and produced comparable results for PFOS. The performance of these linear models indicates that they captured useful concentration-related information, but does not establish that the underlying spectral response is entirely linear. The remaining log-scale errors indicate that precise concentration determination requires further improvement. Future work will expand the dataset to include more independently prepared SERS substrates and a broader range of mixture compositions and concentrations, with the aim of improving predictive accuracy and robustness.

For application to environmental water samples, matrix interference remains an important consideration. Full-spectrum multivariate analysis uses information distributed across the Raman fingerprint and may reduce sensitivity to interference affecting individual bands. However, coexisting PFASs and other substances may introduce overlapping features, alter background signals, or affect analyte adsorption. The present results demonstrate binary-mixture prediction under controlled laboratory conditions; selectivity against interferents absent from the calibration dataset remains to be established. Future work will extend calibration to additional environmentally relevant PFASs and representative water matrices, with performance evaluated using independent field samples and LC–MS/MS measurements.

## 4. Conclusions

In conclusion, this study extended our previously established SERS platform with single-photon Raman detection to binary PFOA/PFOS mixture analysis. Single-photon detection provided the spectral readout, while PLSR and Linear SVR extracted concentration information from overlapping spectral features. Under composition-grouped five-fold cross-validation, the models achieved $R^{2}$ values of 0.806–0.866, with Linear SVR improving PFOA prediction and providing comparable performance for PFOS. Both models achieved 100% ±1-level accuracy, meaning that predictions mapped to the correct or an immediately adjacent experimental concentration level. However, concentration-dependent bias and residual variability limited precise quantification, particularly at $10^{-15}$ M, which remains the lowest nominal concentration investigated rather than a validated LOD or LOQ. The main contribution of this work is therefore the extension of the existing detection platform to multivariate mixture analysis. Future work will expand the dataset across independent substrates, mixture compositions, and water matrices, with validation against LC–MS/MS to assess quantitative reliability under environmentally relevant conditions.

## Figures and Tables

**Table 1:** Predictive performance of PLSR and Linear SVR for PFOA/PFOS mixtures, calculated from pooled held-out predictions under composition-grouped five-fold cross-validation. RMSE and MAE are expressed on the $\log_{10}\left[C/\left(\mathrm{mol}\;L^{-1}\right)\right]$ scale.

Analyte	Model	$R^{2}$	RMSE	MAE
PFOA	PLSR	0.813	1.451	1.223
PFOA	Linear SVR	0.866	1.229	0.987
PFOS	PLSR	0.809	1.466	1.243
PFOS	Linear SVR	0.806	1.477	1.199

## Data Availability

The data supporting the findings of this study are available from the corresponding author upon reasonable request.

## Supplementary Materials

The following supporting information can be downloaded at https://www.mdpi.com/article/10.3390/s26216865/s1, Section S1: Model Training and Hyperparameter Selection; Section S2: Raman Acquisition Parameters; Figure S1: Comparison of Solvent Blank and Low-Concentration PFOA and PFOS Spectra; Table S1: Signal-to-Noise Ratios; Table S2: Model Hyperparameters Selected Across Cross-Validation Folds.

## Abbreviations

The following abbreviations are used in this manuscript:

AOTF	Acousto-optic tunable filter
AgNP	Silver nanoparticle
EDFA	Erbium-doped fiber amplifier
LC-MS/MS	Liquid chromatography–tandem mass spectrometry
MAE	Mean absolute error
PAH	Poly(allylamine hydrochloride)
PFASs	Per- and polyfluoroalkyl substances
PFOA	Perfluorooctanoic acid
PFOS	Perfluorooctane sulfonate
PLS/PLSR	Partial least squares/partial least squares regression
RMSE	Root mean square error
SERS	Surface-enhanced Raman spectroscopy
SPD	Single-photon detector
SVR	Support vector regression
WDM	Wavelength-division multiplexing
