# Supplementary material for: Quantitative Analysis of Perfluorooctanoic Acid and Perfluorooctane Sulfonate Mixtures Using 3D Surface-Enhanced Raman Spectroscopy with Single-Photon Detection
Source: Sensors (Basel). 2026 Oct 30;26(21):6865. doi: 10.3390/s26216865 (PMC13766081; doi:10.3390/s26216865)
Supplement: Supplementary file 1 [file sensors-26-06865-s001.zip › sensors-4637846-supplementary.pdf]

Supplementary Materials

S1. Model training and validation

Nested GroupKFold cross-validation was used, with five outer folds for performance evaluation and four inner folds for hyperparameter selection. Groups were defined by the 16 PFOA/PFOS concentration combinations, with all spectra from each combination kept together in both validation loops.

Hyperparameters were selected separately for each analyte by maximizing the mean inner-fold R^2. The number of PLSR components was searched from 1 to 10. For Linear SVR, C was selected from $\{0.01, 0.1, 1, 10, 100\}$ and ϵ from $\{0, 0.01, 0.05, 0.1, 0.3\}$. Linear SVR used `max_iter=20000`, `dual="auto"`, and `random_state=42`.

StandardScaler was included within each model pipeline and fitted only to the corresponding training data. After hyperparameter selection, the model was refitted on the complete outer training set and evaluated on the held-out groups. Performance metrics were calculated from the pooled outer-fold predictions. The parameters selected in each outer fold are listed in Table S2.

S2. Raman acquisition parameters

Raman measurements were performed using 775 nm pulsed excitation at an average power of 12 mW at the sample and a repetition rate of 50 MHz. The beam was focused using a 100× objective with a numerical aperture of 0.8. The AOTF was scanned over 775–900 nm, corresponding approximately to Raman shifts of 0–1800 cm^{-1}, with a nominal Raman-shift step of 3 cm^{-1}. Photon counts were accumulated for 0.5 s at each spectral sampling point, giving a total acquisition time of 300.5 s per spectrum, including scan overhead. Photon arrival times were recorded with a time-tagger bin width of 50 ps and a synchronization delay of 5 ns.

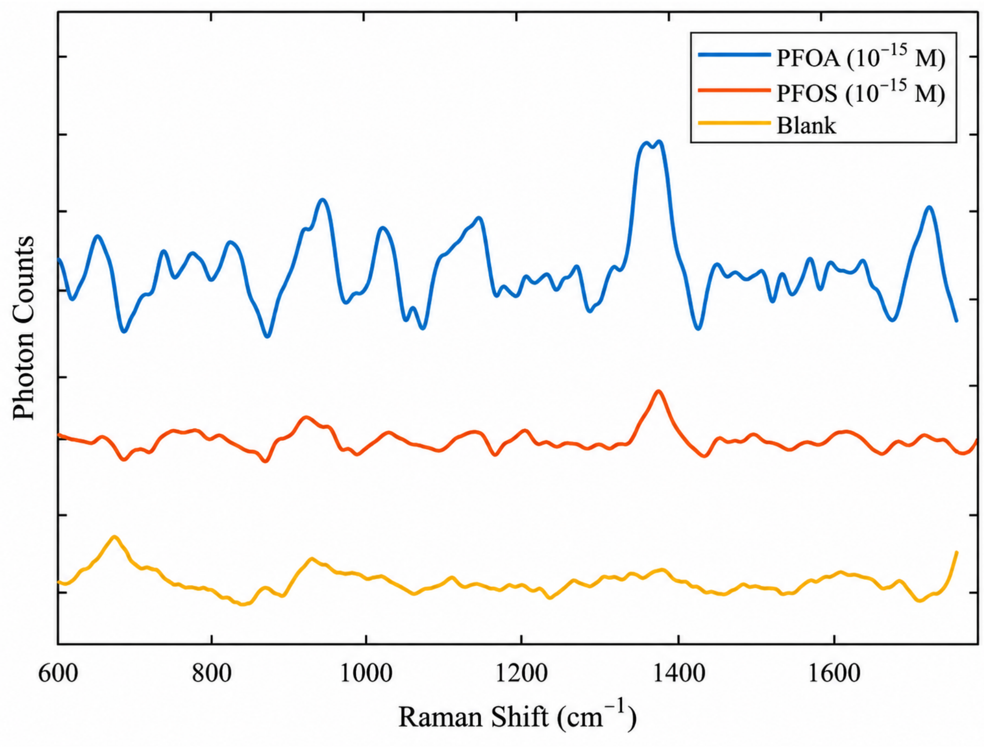

Figure S1: Comparison of solvent blank and low-concentration PFOA and PFOS spectra.

Table S1: Preliminary signal-to-noise ratio (S/N) estimates for PFOA, PFOS, and solvent blank spectra.

Sample	Concentration (M)	Peak position (cm^{-1})	Net peak height	Noise SD	S/N
PFOA	10^{-15}	925.9	658.99	64.31	10.25
PFOA	10^{-15}	1364.4	1069.90	64.31	16.64
PFOS	10^{-15}	921.9	197.86	31.18	6.35
PFOS	10^{-15}	1354.4	335.44	31.18	10.76
Blank	—	925.9	184.56	48.10	3.84
Blank	—	1354.4	73.45	48.10	1.53

S/N values were calculated from the averaged spectrum for each condition.

Table S2: Hyperparameters selected in each outer fold of nested GroupKFold cross-validation. Parameters were selected separately for each analyte by maximizing the mean inner-fold R^2.

**Analyte**	**Parameter**	**Fold 1**	**Fold 2**	**Fold 3**	**Fold 4**	**Fold 5**
PFOA	PLSR components	10	9	9	4	4
	Linear SVR C	1	1	1	10	1
	Linear SVR ϵ	0	0.3	0	0.3	0.01
PFOS	PLSR components	7	5	8	9	7
	Linear SVR C	10	1	10	1	1
	Linear SVR ϵ	0.3	0.3	0.3	0.1	0.3
